# The Genomic Shock Hypothesis: Genetic and Epigenetic Alterations of Transposable Elements after Interspecific Hybridization in Plants

**DOI:** 10.3390/epigenomes8010002

**Published:** 2023-12-27

**Authors:** Carlos de Tomás, Carlos M. Vicient

**Affiliations:** Centre for Research in Agricultural Genomics, CRAG (CSIC-IRTA-UAB-UB), Campus UAB, Cerdanyola del Vallès, 08193 Barcelona, Spain

**Keywords:** transposable element, retrotransposon, MITE, hybridization, genomic shock, epigenetics, DNA methylation, siRNA, stress

## Abstract

Transposable elements (TEs) are major components of plant genomes with the ability to change their position in the genome or to create new copies of themselves in other positions in the genome. These can cause gene disruption and large-scale genomic alterations, including inversions, deletions, and duplications. Host organisms have evolved a set of mechanisms to suppress TE activity and counter the threat that they pose to genome integrity. These includes the epigenetic silencing of TEs mediated by a process of RNA-directed DNA methylation (RdDM). In most cases, the silencing machinery is very efficient for the vast majority of TEs. However, there are specific circumstances in which TEs can evade such silencing mechanisms, for example, a variety of biotic and abiotic stresses or in vitro culture. Hybridization is also proposed as an inductor of TE proliferation. In fact, the discoverer of the transposons, Barbara McClintock, first hypothesized that interspecific hybridization provides a “genomic shock” that inhibits the TE control mechanisms leading to the mobilization of TEs. However, the studies carried out on this topic have yielded diverse results, showing in some cases a total absence of mobilization or being limited to only some TE families. Here, we review the current knowledge about the impact of interspecific hybridization on TEs in plants and the possible implications of changes in the epigenetic mechanisms.

## 1. Transposable Elements

Transposable elements (TEs) are DNA sequences with the ability to change their position in the genome or to create copies of themselves in other positions in the genome [1]. TEs are major components of many plant genomes. For example, TEs comprise more than 90% of the wheat genome [2]. There is a high correlation between TE content and overall nuclear genome size across the angiosperms [3]. Dramatic changes in genome sizes between closely related species have been described due to the TE expansions and contractions [4,5].

Numerous studies support the importance of TEs in the structure and functionality of the genome and their influence on the evolution of plants as an important source of genetic variability [6,7]. There are several examples of mutations and other genetic variations determined by the activity of TEs, for example, their insertion near or within promoters, intronic regions, or enhancer regions, some of them having important consequences in the domestication of plants [8]. 

TEs can be classified into two major classes. Class I elements transpose via an RNA intermediate and through the “copy-and-paste” mechanism and can be further classified in LTR (long terminal repeat) and non-LTR [9], which include Long Interspersed Nuclear Elements (LINEs) and Short Interspersed Nuclear Elements (SINEs). Class II elements or DNA transposons employ the “cut-and-paste” transposition mechanism [10] and can be subdivided into TIR (terminal inverted repeats) and rolling circle Helitrons [9,11]. Another interesting type of DNA transposons highly abundant in the plant genomes are the Miniature Inverted-Repeat Transposable Elements (MITEs), which are non-autonomous deletion derivatives of full-length DNA transposons [1]. Another less studied repetitive element, also present in plant genomes, is the endogenous viruses which are sequences derived from viruses that have integrated into the nuclear chromosomes [12].

## 2. Epigenetic Control of Transposable Elements

TEs have the potential to make copies of themselves at new sites in the genome which can generate mutations and chromosomal rearrangements. Although the progressive accumulation of random deleterious mutations (replication-based errors and point mutations) and the illegitimate or ectopic recombination between copies of the same TE family lead to the inactivation or removal of some TEs, this can take thousands of years, especially if an active TE family has accumulated a high copy number [5,13]. For this reason, cells have developed an array of defense mechanisms that, although they do not destroy TEs permanently, do inactivate them using epigenetic mechanisms [14]. Although the epigenetic inactivation is potentially reversible, leading to a dynamic balance between TE suppression and reactivation, in plants, the epigenetic repression is usually trans-generational and results in subsequent generations of TE repression [15].

### 2.1. Epigenetic Silencing of TEs: Initiation

The initiation of TE silencing in plants involves triggering the RNA-directed DNA methylation (RdDM) pathway [13,16]. An active family can generate multiple copies as long as it does not happen that one of its copies acquires the property of inducing the RdDM pathway, causing inhibition of the entire family. There are different reasons because a copy could become a poison element. For example, it could be inserted downstream of a gene promoter in an antisense position generating double-stranded RNAs, as in the case of the maize Mu killer element [17]. In other cases, single elements can trigger silencing by transposing into other copies of the same family or if they contain aberrant structures such as inverted duplications that, when transcribed, produce double-stranded RNAs (dsRNAs) that trigger the production of small interfering RNAs [18,19]. These triggering TEs act as targets that can be recognized by Pol IV and transcribed into short single-stranded RNAs of 30–40 nucleotides (nt) that RNA DEPENDENT RNA POLYMERASE 2 (RDR2) will copy into dsRNAs that will then be processed into 24 nt small interfering RNAs (siRNAs) by DICER-LIKE 3 (DCL3) [20] and can then be incorporated into ARGONAUTE 4 (AGO4) or 6 (AGO6) to target the scaffold transcripts generated from Pol V, leading to de novo recruitment of DNA methyltransferase REORGANIZED DOMAINS METHYLTRANSFERASE 2 (DRM2) to the original or homologous sequences to trigger methylation in all three cytosine contexts (CG, CHG, and CHH) [21]. The only condition is that the triggering copy has enough sequence complementarity with the rest of the TE family members to keep them repressed. The canonical RdDM pathway mainly affects TEs located in places where chromatin is more accessible (short TEs and ends of long TEs close to genes) and would serve to prevent the spread of euchromatin from genes to neighboring TEs [22]. For TEs located in sites where heterochromatin is profoundly inaccessible, methylation is catalyzed by METITRANSFERASE 1 (MET1) for CG, CHROMOMETHYLASE 3 (CMT3) for CHG, and CHROMOMETHYLASE 2 (CMT2) for CHH, and generally depends on DECREASE OF DNA METHYLATION 1 (DDM1) [22].

Another proposed mechanism to initiate TE silencing is based on the existence of copies in which the coding regions have non-optimal codon usage. This would cause their mRNAs to suffer frequent stalling in the ribosomes, which would induce numerous RNA truncations. These truncated RNAs would be more prone to be attacked by RNA DEPENDENT RNA POLYMERASE 6 (RDR6) [23]. RDR6 seems to be involved in a non-canonical RdDM mechanism [24]. TE RNA polymerase II (Pol II) transcripts can be processed by RDR6 and DICER-LIKE 2 (DCL2) or 4 (DCL4) into 21 or 22-nt siRNAs and then loaded into AGO6 via a Pol V scaffold transcript to activate the initial methylation of the TE family and then canonical RdDM takes over the full methylation. 

### 2.2. Epigenetic Silencing of TEs: Maintenance

Once the initial methylation of the TE is established, DNA methylation is maintained by multiple pathways and methyltransferases at CG, CHG, and CHH sequence contexts [25]: MET1 to maintain CG cytosine methylation, CMT3 to maintain CHG methylation and CHH methylation is maintained by persistent de novo methylation of DRM2 via the RdDM pathway (in regions of relatively open chromatin) or by CMT2 in conjunction with H3K9me2 (in deep heterochromatic regions). TE silencing is subject to canonical RdDM to reinforce its silencing [16] and the silenced state of TEs is hereditarily transmitted over generations if nothing alters it.

### 2.3. Epigenetic Silencing of TEs: Loss

There are genetic and environmental situations in which TE families that have been silenced for generations can escape silencing and transpose again [26]: biotic and abiotic stresses [27,28,29,30], tissue culture [31], inbreeding [32] or interspecific hybridization [33,34]. Little is known about why and how silencing mechanisms are not functional for a subset of species and TE families while remaining active for other TEs in the genome. In general, reactivated TEs are re-silenced again after a period of activity, generating bursts of TE amplification [35], but the time in which re-silencing occurs may depend on multiple factors. 

TEs located in relatively open chromatin regions, such as those close to genes, are more susceptible to transcriptional activation while those located in condensed heterochromatic regions with the lowest recombination rates, which are usually the most abundant in the genome, are heavily methylated and modified with repressive histones, meaning that they are deeply silenced and the maintenance of silencing of these TEs is very stable, even in the presence of stress [21,36,37]. In this way, TEs that are inserted in regions close to genes (such as MITEs) have an advantage that makes them more prone to reactivation.

Through a mechanism called DNA acquisition, some viruses can incorporate host DNA sequences that can add new functions to the virus, such as resistance to silencing [38,39]. In the same way, as in viruses, some TEs seem to have developed systems to avoid being silenced. For example, an anti-silencing function has been demonstrated for the VANC protein encoded by the Arabidopsis transposon VANDAL [40], the TnpA protein encoded by the maize En/Spm transposon [41], and the HDP1 and HDP2 proteins of the Harbinger transposon in Arabidopsis [42]. Although similar mechanisms have not yet been described in plant retrotransposons, it is known that many of them contain conserved ORFs of unknown function [43], in some cases showing high levels of transcription [44], which could encode proteins with anti-silencing functions. In a similar way, TEs can also acquire DNA sequences that act as enhancers to promote the expression of the TE during specific conditions.

## 3. Changes in the Epigenetic Silencing of the TEs in Plant Interspecific Hybrids

Interspecific hybridization is a very common process in plants [45] and has great relevance in their evolution since it can have important consequences on the phenotype and can even give rise to new species [46]. Interspecific hybridization has its application in breeding as hybridization with wild relatives is frequently used to expand the variability of cultivated species [47]. Hybridization can increase mutation rates, increase chromosomal rearrangements, and induce epigenetic changes, including changes in DNA methylation and small RNA populations [48,49]. These alterations may result in changes in genome size (up or down) [50] and in the activation and mobilization of some families of TEs (burst amplification) [51] and can be so extensive that they have sometimes been called genomic shock [52]. However, an increasing number of cases have been reported in which the interspecific crosses do not have consequences, or no such great consequences. Next, we will compile the reported cases of the effect of interspecific hybridization over the TEs in plants (Table 1). We have also included the cases of allopolyploidization, a type of hybridization between two species in which the hybrids acquire the complete diploid chromosome complements of the two parents. 

### 3.1. Zea

Based on her work in maize, Barbara McClintock predicted in the 80s that hybridization in plants might activate quiescent transposons and result in genome restructuring [53]. However, Anderson et al. (2019) [54] analyzed maize TE expression and found that most expressed TE families do not show differential expression in hybrids and there are more families that are expressed much lower in the hybrid than in both parents that are expressed higher. Guo et al. (2013) [55] found differences in the accumulation of some TE-encoded proteins in the hybrid Zong3/87-1 with respect to their parents. Barber et al. (2012) [56] analyzed how siRNA populations vary between two maize inbred lines (B73 and Mo17) and their hybrid in the shoot apex and the developing ear (high percentages of these siRNAs derive from retrotransposons) and found that the small RNA levels are altered in the hybrids. In the same hybrid, Liu et al. (2023) [57] found that there are regions where methylation decreases after hybridization despite the production of small RNAs.

### 3.2. Helianthus

Baack et al. (2005) [50] estimated the nuclear DNA content in three homoploid hybrid *Helianthus* species (*H. anomalus*, *H. deserticola*, and *H. paradoxus*) and the parental species (*H. annuus* and *H. petiolaris*) and the hybrid-derived species have 50% more nuclear DNA than the parental species. These increases are due basically to the accumulation of certain families of Ty3/gypsy retrotransposons in the hybrid [58,59,60] although some Ty1/copia retrotransposon families also contributed to a lesser extent [61]. Interestingly, the retrotransposon families that proliferated in the hybrids are the ones that show a higher transcriptional activity not only in the hybrid but also in the parents [62]. However, synthetic hybrids between *H. annuus* and *H. petiolaris* showed no increase in genome size, retrotransposon copy numbers, or transcription [50,62,63,64]. 

### 3.3. Capsella

Ågren et al. (2016) [65] examined the TE content in the allotetraploid *Capsella bursa-pastoris*, comparing it with the two parental diploid species, *C. grandiflora* and *C. orientalis*. They found no significant differences in the total numbers of TEs, but when centromeric regions are excluded (they constitute most of the TE content of these genomes), they found evidence of a significantly higher abundance of retrotransposons in *C. bursa-pastoris* compared with *C. grandiflora* and *C. orientalis*. However, in a similar more recent work using massive sequencing, no signs of large-scale TE-reactivation in synthetic diploid hybrids, autotetraploids, or allotetraploids were found [66].

### 3.4. Aegilops

Senerchia et al. (2015) [67] used reciprocal F1 hybrids between three *Aegilops* species and observed copy number increase in the hybrids of some retrotransposon families and significantly higher DNA methylation in the retrotransposons that authors suggest is an immediate response to support hybrid viability. In another study, Senerchia et al. (2016) [68] analyzed retrotransposon behavior in hybrid populations of *A. geniculata* and *A. triuncialis* and observed that some TE families are activated in the hybrid, especially those that have been recently active in one of the parents, and that activation is different according to the species acting as male or female. Active TEs have also proliferated in the *A. markgrafii* genome, a species derived from hybrid speciation [69] and in artificial intraspecific hybrids of *A. speltoides* [70]. 

### 3.5. Arabidopsis

In the synthetic allopolyploid *Arabidopsis suecica* (*A. thaliana* × *A. arenosa*), Madlung et al. (2005) [71] found an enhanced, but limited, transcriptional and transpositional activity of both DNA and RNA TEs compared to the parental lines. The retrotransposon Athila is expressed in the hybrids, but only paternal (and not maternal) copies are expressed [33]. The En-Spm-like transposon Sunfish displayed an enhanced transcriptional activity in the hybrid and this transcription was correlated with a reduction in cytosine methylation of the element [71]. The mobility of the sunfish transposon was detected in *A. suecica* synthetic allotetraploids [72]. Ha et al. (2009) [73] studied the presence of small RNAs in natural and re-synthesized allotetraploid *A. suecica* and their results showed that the TE-associated siRNA population underwent rapid changes in F1, becoming stable in the next generations. 

In a synthetic allopolyploid (*A. thaliana × A. lyrata*), changes in the degree of DNA methylation were observed, but no evidence of increased mobility of TEs was obtained [34,74,75].

*A. thaliana* accessions Columbia and Landsberg erecta, with their reciprocal hybrids, were used to analyze the DNA methylation and small RNA profiles. The small RNAs that overlap TEs are highly represented in the F1 but different studies found that they do not differ from the parents [76,77,78], although Groszmann et al. (2011) [79] found a reduction in the siRNAs in the hybrids. With respect to DNA methylation, both hybrids displayed increased DNA methylation across their entire genomes, especially in TEs [80].

Rigal et al. (2016) [81] studied the effects of TEs on crossing a mutant defective in the maintenance of DNA methylation (met1) with Col-0 wild-type individuals. The met1 mutants show over 2000 re-activated TEs. The F1 hybrid plants showed a substantial increase in DNA methylation in TEs, especially those located in pericentromeric regions, but transcriptional re-silencing was not complete in the F1 hybrids which show an increased TE transcriptional activity with respect to the wild-type parent.

### 3.6. Arachis

Tang et al. (2022) [82] detected the mobilization of *AhMITE1* induced by hybridization of *Arachis duranensis* with *A. ipaensis*. 

### 3.7. Brassica

No evidence of TE mobility in response to allopolyploidization was found in *Brassica* natural species [83,84]. Accordingly, the Bot1 CACTA element, originally from *B. oleracea*, is C genome-specific in the allopolyploid [84]. Biased patterns of siRNA density and expression among subgenomes were observed in allopolyploid *B. napus* and significant differences in overall TE composition and densities near genes were shown to exist in each of the subgenomes [85].

The situation is more complex in the synthetic allopolyploids. No evidence of TE mobility in response to allopolyploidization was found in synthetic allopolyploids by Lukens et al. (2006) [86]. However, active TEs were found in a re-synthesized *B. napus*, including LTR retrotransposons, DNA transposons, and non-autonomous TEs [87,88]. Activation seems to be higher in LTR retrotransposons and in the first two generations after hybridization [89]. These activations are accompanied by higher transcription levels in some of the TE families [90]. Important changes in DNA methylation were observed in the synthetic allopolyploids including TEs [86,91], in which hypermethylation is more frequent than hypomethylation [92]. Shen et al. (2017) [93] found in the F1 an increase in the levels of TE-associated siRNA and the levels of DNA methylation. 

Zhang et al. (2023) [94] studied the effects of allotetraploidization between *B. rapa* and *B. oleracea* during eight generations using mRNA-seq and bisulfite-seq. They found that the differences in TE load and/or DNA methylation levels near genes were not negatively associated with subgenome dominance. 

These apparently contradictory results may be a consequence of the use of different hybridization events but also of the use of different analytical techniques.

### 3.8. Camellia

Zhang et al. (2018) [95] detected an increase in the transcription of several TEs in the F1 hybrid *Camellia azalea* × *C. amplexicaulis*. They also detected changes in gene expression in genes related to RNA-directed DNA methylation and histone methylation. 

### 3.9. Dactylorhiza

Eriksson et al. (2022) [96] studied the TE content in five naturally produced allotetraploids derived from *Dactylorhiza fuchsii* and *D. incarnata*. Interestingly, *D. incarnata* has a much larger genome than *D. fuchsii* due to a major content of TEs. In the allopolyploids, the genome size is additive in the younger ones but appears enlarged in the older allopolyploids. These genome expansions are mainly due to sequences derived from MITEs. They deduce that, apart from the MITE-like element, no significant “genomic shock” follows the formation of the allopolyploids.

### 3.10. Gossypium

Zhao et al. (2018) [97] examined the profile of lncRNAs in *Gossypium arboreum* and *G. raimondi* and their F1 hybrid. They found that the non-coding transcriptome undergoes tremendous variation after hybridization and many of the activated lncRNAs are transcribed from de-methylated TE regions, especially from LINEs. 

### 3.11. Lotus

Fukai et al. (2022) [98] investigated the transposition of LTR retrotransposons in recombinant inbred lines (RILs) of *Lotus japonicus* and detected that six LTR retrotransposon families were activated and transposed in 78% of the investigated RILs. They also detected an epigenetic de-repression of LORE1a LTR retrotransposon in the F1 and also across generations, indicating long-term effects of hybridization in the TE activity. 

### 3.12. Nicotiana

In *Nicotiana sylvestris* × *N. tomentosiformis* hybrid (synthetic *N. tabacum* allotetraploid), a significant increase of Tnt1 LTR retrotransposon copy number was observed derived from maternal elements [99]. In a similar study, but using three synthetic allotetraploids (*N. arentsii*, *N. rustica*, and *N. tabacum*) Mhiri et al. (2019) [100] compared the dynamics of six TEs in these allopolyploids, their diploid progenitors and in corresponding synthetic hybrids, and found that in young *Nicotiana* allopolyploids, TE activation occurred during the first generations of the allopolyploids.

### 3.13. Oryza

Hybrids between *Oryza sativa* and *Zizania latifolia* show an increase in the copy number of some LTR retrotransposon families [101] and MITEs [102]. Interestingly, transcriptional activation of TEs and extensive DNA methylation changes (including in TEs) have been reported in this hybrid [103,104]. On the other hand, in *Oryza sativa* × *Oenothera biennis* hybrids, the mobilization of mPing and three LTR retrotransposons have been detected correlated to changes in DNA methylation [105].

### 3.14. Poa

Benson et al. (2023) [106] studied the LTR retrotransposon content of the allotetraploid *Poa annua* and compared it with the parents *Poa infirma* and *P. supina*. They observed a bias in transposon movement from one subgenome to the other. They hypothesize that it is at least partially driven by differences in the subgenome’s ability to repress TEs post-transcriptionally due to differences in the heterochromatin, euchromatin distribution, or DNA methylation. They also found the existence of important differences between individuals in the TE presence.

### 3.15. Populus

Usai et al. (2020) [107] examined the TE mobilization in *Populus canadensis*, the interspecific hybrid of *P. deltoides* and *P. nigra*. The poplar hybrid showed differences in the abundance of certain LTR-retrotransposon families compared to the parents. They also detected a relatively high number of hemizygous LTR-retrotransposon copies (present in a locus in one chromosome, but absent in the same locus in the homologous chromosome). At least, a part of these hemizygous elements is a consequence of the production of new copies of LTR-retrotransposons subsequently to the interspecific hybridization indicating that LTR-retrotransposon mobilization occurred during the first clonal generations of the interspecific cross. These hemizygous elements are only restricted to certain lineages as the TAR/Tork elements. The TAR/Tork elements are the most recently active LTR retrotransposons in *P. trichocarpa* [108]. Transcriptomic data showed a generally low expression level of LTR retrotransposons in the hybrid and the parents, but some specific families showed a higher transcription in the hybrid. 

### 3.16. Solanum

Raza et al. (2017) [109] performed a comparative analysis of the DNA methylation patterns in *Solanum lycopersicum*, *S. pimpinellifolium*, and their reciprocal hybrids and found that the reciprocal hybrids had lower levels of DNA methylation in LTR retrotransposons than their parents. Gantuz et al. (2021) [110] evaluated the proliferation of LTR retrotransposons in a diploid hybrid between *S. tuberosum* and *S. kurtzianum* and allotetraploid lines derived from this hybrid. They found that some LTR retrotransposon families are activated principally in the hybrid. Previously, Marfil et al. (2006) [111] showed an alteration in the methylation status of the hybrid with respect to the parents, although they did not determine which type of DNA sequences were mainly affected. Paz et al. (2015) [112] studied the effects of the hybridization between *S. kurtzianum* and *S. microdontum* in the activity and DNA methylation of Tnt1 and Tto1 retrotransposons. They observed moderate mobility and a demethylation of both LTR retrotransposons in the hybrid compared with the parents. In general, in the *Solanum* species hybridization seems to activate certain TEs accompanied by a reduction in DNA methylation.

### 3.17. Triticum

Alterations in the DNA methylation status in F1 hybrids and allopolyploid species from the wheat (*Aegilops* and *Triticum*) group were found in both repetitive DNA sequences, such as LTR retrotransposons, and in low-copy DNAs [113] (Shaked et al., 2001).

Higher transcriptional activity of the Wis2-1A LTR retrotransposon have been observed in synthetic allotetraploid wheat compared with its diploid parental lines (*A. sharonensis* × *T. monococcum*) [114] affecting the expression of adjacent genes due to the production of readout transcripts [115]. Banouh et al. (2023) [116] also found increased transcription in TE families in the hybrid, although the differences were only observed in three families and were not high.

Kraitshtein et al. (2010) [117] analyzed the behavior of the Veju elements (TRIM) in the first generations of a newly formed allohexaploid (*T. turgidum* × *A. tauschii*). They found that while DNA hypomethylation was significantly predominant in the first three generations, DNA hypermethylation became predominant in the subsequent generations. On the other hand, many Veju elements were deleted in the first generation but, in subsequent generations, their numbers increased with most new Veju insertions produced in the second generation. In contrast to Veju, the analysis of three DNA transposon elements, Balduin (CACTA), Apollo (MuDR), and Thalos (MITE), in the same samples showed that they underwent massive DNA hypermethylation in the first four generations [118].

Kenan-Eichler et al. (2011) [119] studied the presence of small RNAs after *A. tauschii* × *T. turgidum* hybridization and allopolyploidization, in special, small RNAs corresponding to TEs. They found that the percentage of siRNAs corresponding to TEs strongly decreased upon allopolyploidization, but not upon hybridization. Moreover, Kirov et al. (2020) [120] found that some LTR retrotransposon-related transcripts originated from autonomous LTR retrotransposons are accumulated during the early stages (10 days post anthesis) of seed development, most of them encoding for GAG proteins. Experiments conducted with similar samples showed that siRNA densities at TE-associated regions vary between each of the three subgenomes being higher in the D genome which may account for biased repression of the D-TEs (*A. tauschii*) [121].

Finally, Bento et al. (2008) [122] detected genomic DNA sequence rearrangements associated with LTR retrotransposons in the triticale genome (*T. aestivum* × *Secale cereale*) but no transposition bursts were reported.

### 3.18. Vitis

Cadle-Davidson and Owens (2008) [123] studied the copy numbers of the Ty3-gypsy-type retrotransposon Gret1 in different species of the *Vitis* genus as well as in hybrids. They found that the highest Gret1 copy numbers are observed in hybrids. 

### 3.19. Yucca

Heyduk et al. (2021) [124] studied the effects of hybridization in *Yucca gloriosa*, a homoploid hybrid resulting from a cross between *Y. aloifolia* and *Y. filamentosa*. TE abundance in the hybrid was intermediate to the parental species except for one LTR retrotransposon family whose abundance was higher relative to both parents. They did not detect significant changes in TE transcription.

### 3.20. Cajanus

Junaid et al. (2018) [125] studied the consequences of the cross between two pigeon pea lines differing in male fertility. Overall, they observed a higher DNA methylation level in the hybrid, including TEs, concluding that there is no genomic shock. However, they found several differentially methylated regions (DMRs), many of them located in TEs, and several of them negatively associated with gene expression in the hybrid. 

### 3.21. Hieracium

Zagorski et al. (2020) [126] studied the diploid F1 synthetic and the natural triploid hybrids of *Hieracium intybaceum* and *H. prenanthoides*. No TE bursts were detected, but the hybrid displayed an overabundance of endogenous pararetrovirus clusters not observed in synthetic hybrids.

### 3.22. Mimulus

Edger et al. (2017) [127] examined TE methylation in a natural allopolyploid (*Mimulus peregrinus*), a resynthesized interspecies triploid hybrid (*M. robertsii*), a resynthesized allopolyploid (*M. peregrinus*), and progenitor species (*M. guttatus* and *M. luteus*). They found significant decreases in the TE CHH methylation levels in the F1 hybrid and a re-establishment of CHH methylation levels in subsequent generations returning to near parental levels. However, the return was not equal in the subgenomes, and found that the recessive subgenome had returned to near parental CHH methylation levels, while the dominant subgenome retained CHH methylation below parental values. These differences are correlated with a dominant subgenome expressed genes.

### 3.23. Prunus

De Tomás et al. (2022) [128] analyzed the F1 hybrid between *Prunus persica* and *P. dulcis* and found that it did not result in important changes in the regulation of TEs. The levels of TE transcription are not increased in the hybrid and the expression of genes potentially involved in the regulation of the TE activity and DNA methylation dynamics is not altered except for a reduced expression in the hybrid in two genes encoding for an RNA-dependent RNA polymerases similar to the RDR1 protein from Arabidopsis. There are no major differences in the TE methylation levels but they found some DMRs that overlap with certain families of LTR retrotransposons that are demethylated in the hybrid compared to peach only in the CHG context but without an associated transcriptional reactivation. Using different parents of the same species, D’Amico-Willman et al. (2022) [129] observed that the overall levels of methylation did not differ in the hybrid, although they identified DMRs in each methylation context, some of them associated with TEs. However, these DMRs vary in different individuals of the F1. Overall, no genomic shock was observed in the crosses between *Prunus* species, although in some cases, there are punctual differences in DNA methylation associated with TEs.

### 3.24. Spartina

A limited transpositional TE activation has been found in allopolyploids and hybrids of *Spartina*, but without extensive transposition bursts [130]. A more recent study found higher levels of transcriptome repatterning following neopolyploidy [131]. Low levels of mobilization and changes in the TE methylation status have been found in allopolyploids and hybrids of *Spartina*, but without extensive transposition bursts [130,131,132]. In addition, Cavé-Radet et al. (2019) [133] found a differential expression of TE-related small RNAs following recent speciation in polyploid *Spartina*. These results reinforce the view that *Spartina* allopolyploids and hybrids suffer a limited activation of TEs that seems to be epigenetically silenced quickly.

### 3.25. Sorghum

*Sorghum halepense* is an allopolyploid species formed by hybridization between diploid *S. bicolor* and *S. propinquum*. Kuo et al. (2021) [134] compared the repeat profiles of *S. bicolora* and *S. halepense* and they did not detect large-scale amplification or reduction of repeat sequences in the allotetraploid with respect to *S. bicolor*.

**Table 1:** Studies carried out on the effect of hybridization on the activity of transposable elements.

Species	Hybrid Type	Genomic Shock	Transcription Alterations	DNA Methylation ^1^ Alterations	Description	References
*Capsella bursa-pastoris* (*C. grandiflora* × *C. orientalis*)	Allotetraploid	Yes	-	-	Higher number of TEs only in gene-rich chromosome arms with no important global differences.	[65,66]
*Helianthus* spp.	Hybrid Natural & synthetic	Yes	Yes	-	Ancient hybrids have more DNA than parents due to the expansion of certain TE families that are transcriptionally active. Synthetic hybrids do not show increases in genome size.	[8,50,59,60,61,62,63,64]
*Zea mays*	Hybrid	Yes	-	-	Alterations in siRNAs and DNA methylation.	[56,57]
	Hybrid	Yes	-	-	Differences in TE protein accumulation.	[55]
	Hybrid	Yes	Both	-	Most TE families do not show transcriptional differences in the hybrid, but some yes.	[54]
*Aegilops* spp.	Hybrid	Limited	-	Higher	Increase in copy number of some families and increase in DNA methylation in the hybrid.	[67]
*Aegilops geniculata* × *A. triuncialis*	Hybrid	Limited	-	-	Activation of some retrotransposon families.	[68]
*Aegilops markgrafii*	Allotetraploid	Limited	-	-	Activation of some TE families.	[69]
*Aegilops sharonensis* × *Triticum monococcum*	Allohexaploid	Limited	Yes	-	Transcriptional activation of some retrotransposon families.	[114,115]
*Aegilops speltoides*	Hybrid	Limited	-	-	Activation of some TE families.	[70]
*Arabidopsis suecica* *(A.thaliana × A. arenosa)*	Allotetraploid Natural & synthetic	Limited	Yes	-	Limited higher transpositional activity of TEs in the hybrid. Changes in siRNA population.	[33,71,72,73]
*Arachis duranensis ×* *A. ipaensis*	Allotetraploid	Limited	-	-	Mobilization of AhMITE1.	[82]
*Brassica napus*	Allotetraploid synthetic	Limited	Yes	Higher	Activation of some families is associated with changes in DNA methylation and siRNA contents in some cases and no activation in others.	[86,87,88,89,90,91,92,93,94]
*Camellia azalea* × *C. amplexicaulis*	Hybrid	Limited	Higher	-	Increase in TE transcription.	[95]
*Dactylorhiza*	Allotetraploids	Limited	-	-	Increase in genome size due to the activity of an MITE family.	[96]
*Gossypium arboreum* × *G. raimondi*	Hybrid	Undeter-mined	-	-	Many lncRNAs are activated in the hybrid corresponding to LINEs.	[97]
*Lotus*	RIL population	Limited	-	Lower	Mobilization of some retrotransposons associated with demethylation, but does not seem to affect all TE families.	[98]
*Nicotiana arentsii*, *N. rustica*, *N. tabacum*	Allotetraploid	Limited	-	-	Increase in copy number of some retrotransposon families.	[99,100]
*Oryza sativa* × *Oenothera biennis*	Hybrid	Limited	-	Altered	Mobilization of some TEs and changes in DNA methylation.	[105]
*Oryza sativa ×* *Zizania latifolia*	Hybrid	Limited	Yes	Altered	Increase in some TEs copy numbers and transcription. Changes in DNA methylation.	[101,102,103,104]
*Poa annua* (*P. infirma × P. supina)*	Allotetraploid	Limited	-	-	Differences in TE content and distribution between subgenomes and between individuals.	[106]
*Populus canadiensis* (*P. deltoides* × *P. nigra*)	Allotetraploid	Limited	Higher	-	Differences in the presence of new copies and the transcription of certain retrotransposon families, but not a generalized activation of the TEs.	[107]
*Solanum kurtzianum ×* *S. microdontum*	Hybrid	Limited	-	Lower	Tnt1 and Tto1 retrotransposons have moderate mobility and demethylation in the hybrid.	[112]
*Solanum tuberosum* × *S. kurtzianum*	Hybrid Allotetraploid	Limited	-	-	Activation of certain TE families.	[110]
*Triticum aestivum* × *Secale cereale*	Allohexaploid	Limited	-	-	DNA sequence rearrangements associated with TEs.	[122]
*Triticum turgidum* × *Aegilops tauschii*	Hybrid Allohexaploid	Limited	Yes	Altered	Changes in transcriptional activity and DNA methylation in some TE families.	[116,117,118,119,120,121]
*Vitis*	Hybrids	Limited	-	-	Increase in Gret1 LTR-retrotransposon copy number in hybrids.	[123]
*Yucca aloifolia* × *Yucca filamentosa*	Hybrid	Limited	Similar	-	No significant changes in TE abundance or transcription. Only one LTR retrotransposon family has more abundance in the hybrid.	[124]
*Arabidopsis thaliana* × *Arabidopsis lyrata*	Allotetraploid	No	-	Yes	No increases in TE mobility.	[34,74,75]
*Arabidopsis thaliana* Col-0 × Ler	Hybrid	No	-	Higher	No differences in small RNAs.	[76,77,78,79,80]
*Arabidopsis thaliana* Col-0 × met-1 mutant	Hybrid	No	Lower	Higher	Lower transcription and higher DNA methylation compared to mut1.	[81]
*Brassica napus*	Allotetraploid natural	No	-	-	No differences.	[83,84,85]
*Cajanus cajan*	Hybrid	No	-	Higher	DMRs.	[125]
*Hieracium intybaceum* × *H. prenanthoides*	Hybrid Triploid hybrid	No	-	-	No increase in the TE copy number. Overabundance of endogenous pararetrovirus in triploid hybrids.	[126]
*Mimulus guttatus* × *Mimulus luteus*	Allopolyploid Triploid hybrid	No	-	Lower	Lower DNA methylation in the F1 hybrid returns to the parental levels in few generations, but shows differences between subgenomes.	[127]
*Prunus persica* × *P. dulcis*	Hybrid	No	Similar	Similar	DMRs.	[128,129,135]
Spartina spp	Hybrid Allotetraploid	No	Some	Some	Few new insertions were detected, a limited TE transcriptional increase, and limited DNA methylation changes. Differential expression of TE-related small RNAs.	[130,131,132,133]
*Solanum lycopersicum* × *S. pimpinellifolium*	Hybrid	No	-	Lower	DNA methylation is lower in the hybrid.	[109]
*Sorghum halepense* (*S. bicolor* × *S. ropinquum*)	Allotetraploid	No	-	-	No differences in TE content.	[134]

^1^ DNA methylation in the hybrid respect the parents.

## 4. Conclusions: Genomic Shock?

The merging of two genomes in a hybrid has been proposed to trigger a “genomic shock”, that is, a genome-wide misregulation of the transcriptome and epigenome, disrupting gene regulation and inducing chromosomal rearrangements and the mobilization of TEs [136]. However, according to the results compiled here regarding TEs (Table 1), the existence of genomic shock does not seem to be generalized, being restricted to a few species (8% of the analyzed), while in the vast majority, the activation of TEs in the hybrid is restricted to one or a few families (61%), and, in other cases, to none (31%). Furthermore, we cannot rule out the existence of a certain publication bias, that is, the existence of studies that have not been published because they found no differences between hybrids and parents. If this is true, the percentage of cases in which there is no genomic shock would be even higher. 

It should be noted that our study has some limitations. First, the estimations of TE activity are based on multiple techniques with different coverage and addressing different aspects like transcriptome, DNA methylation, or detection of new insertions. Second, the studies used different types of hybrids including natural and artificial F1 hybrids and allopolyploids, and examined at different times after the hybridization event. Third, the studies use hybrids having very variable parental phylogenetic divergences. Despite this, we believe that some common patterns can be perceived, and the conclusions are based on sufficiently consistent deductions.

So, the phenomenon of genomic shock resulting from hybridization is not universal. How can we explain these differences? Different reasons have been proposed to explain the behavioral variability of TEs in hybrids. One of the proposed reasons is the level of phylogenetic divergence between the parents [100]. According to this hypothesis, the more phylogenetically separated the parents are, the greater the activation of TEs will be. Although this reason cannot be completely ruled out, there are cases in which when crossing varieties of the same species activation of TEs is detected [53] while in others, it is not (Arabidopsis) [34]. In consequence, phylogenetic distance cannot be the only reason.

Another hypothesis that, in our opinion, seems more consistent with the results, is that the response depends on the differences in the content of TEs, especially in potentially active TEs [136]. The differences in TE content between the parents will depend on the time elapsed since the divergence of the species. This differentiation is expected to be greater the longer the separation time, but not necessarily. There are processes that can produce important differences in the potentially active TEs from one plant to another in relatively short time periods. For example, an environmental stress period just after the species differentiation can activate some TE families producing a rapid differentiation in the mobilome [137], horizontal transfer from a different species, human selection, natural mutations, genomic rearrangements, changes in epigenetic controls, etc. Thus, processes can explain why, for example, there are many differences in the content of active elements between varieties in corn [138] and very few between two different species of *Arabidopsis* [138] or *Prunus* [139].

In general, the activation of TEs in hybrids is accompanied by a reduction in DNA methylation in the F1 hybrid which can be general or TE-specific. The DNA demethylation can activate the TE transposition [140]. The methylation status of TEs returns to near parental levels in a relatively short time (a few generations), and siRNAs play an important role in this process [57]. If the active TE content of both genomes is different, the small RNA sequences derived from one parent differ from the other, resulting in an enhanced TE activity depending on which parent contains more active TEs. If the active TE comes through the maternal line, the siRNAs are in the cytoplasm and will inhibit it more effectively. The same if the TEs are present in both subgenomes [33]. However, if the TEs come through the paternal line there will not be as many siRNAs and they will be activated in the F1 to a greater degree.

**Figure 1:**
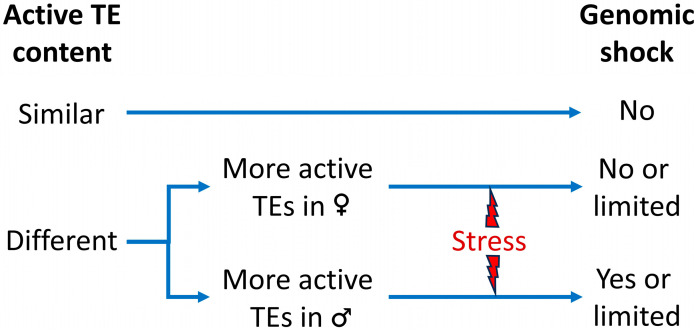
Activation of genomic shock depending on active TE differential contents of males and females and on the presence of stress conditions.

In conclusion, the main point in determining the existence or not of genomic shock seems to be the presence of active TE families in at least one of the parental species, but not the unique ones. For example, in sunflowers, natural hybrids show genome shock while recent hybrids do not, both involving the same parents. It is possible that in the natural hybrids, the hybridization process was accompanied by some type of stress that activated the TEs, while in the artificial ones, this stress was not present [64]. This shows that the activation of genomic shock is a complex process, that involves various factors, and in which the epigenetic regulation of TEs plays a primary role (Figure 1).

## Data Availability

Not applicable.

## References

[1] Feschotte C., Jiang N., Wessler S.R. (2002). Plant transposable elements: Where genetics meets genomics. Nat. Rev. Genet..

[2] Wicker T., Gundlach H., Spannagl M., Uauy C., Borrill P., Ramírez-González R.H., De Oliveira R., Mayer K.F.X., Paux E., Choulet F. (2018). Impact of Transposable Elements on Genome Structure and Evolution in Bread Wheat. Genome Biol..

[3] Lee S.I., Kim N.S. (2014). Transposable elements and genome size variations in plants. Genom. Inform..

[4] Piegu B., Guyot R., Picault N., Roulin A., Saniyal A., Kim H., Collura K., Brar D.S., Jackson S., Wing R.A. (2006). Doubling Genome Size without Polyploidization: Dynamics of Retrotransposition-Driven Genomic Expansions in *Oryza australiensis*, a Wild Relative of Rice. Genome Res..

[5] Vicient C.M., Suoniemi A., Anamthawat-Jónsson K., Tanskanen J., Beharav A., Nevo E., Schulman A.H. (1999). Retrotransposon BARE-1 and Its Role in Genome Evolution in the Genus *Hordeum*. Plant Cell..

[6] Vicient C.M., Casacuberta J.M. (2017). Impact of transposable elements on polyploid plant genomes. Ann. Bot..

[7] Lisch D. (2013). How important are transposons for plant evolution?. Nat. Rev. Genet..

[8] Fambrini M., Usai G., Vangelisti A., Mascagni F., Pugliesi C. (2020). The plastic genome: The impact of transposable elements on gene functionality and genomic structural variations. Genesis.

[9] Mhiri C., Borges F., Grandbastien M.A. (2022). Specificities and dynamics of transposable elements in land plants. Biology.

[10] Wicker T., Sabot F., Hua-Van A., Bennetzen J.L., Capy P., Chalhoub B., Flavell A., Leroy P., Morgante M., Panaud O. (2007). A unified classification system for eukaryotic transposable elements. Nat. Rev. Genet..

[11] Kapitonov V.V., Jurka J. (2007). Helitrons on a roll: Eukaryotic rolling-circle transposons. Trends Genet..

[12] De Tomás C., Vicient C.M. (2022). Genome-wide identification of Reverse Transcriptase domains of recently inserted endogenous plant pararetrovirus (*Caulimoviridae*). Front. Plant Sci..

[13] Liu P., Cuerda-Gil D., Shahid S., Slotkin R.K. (2022). The epigenetic control of the transposable element life cycle in plant genomes and beyond. Annu. Rev. Genet..

[14] Pachamuthu K., Borges F. (2023). Epigenetic control of transposons during plant reproduction: From meiosis to hybrid seeds. Curr. Opin. Plant Biol..

[15] Heard E., Martienssen R.A. (2014). Transgenerational epigenetic inheritance: Myths and mechanisms. Cell.

[16] Matzke M.A., Kanno T., Matzke A.J. (2015). RNA-directed DNA methylation: The evolution of a complex epigenetic pathway in flowering plants. Annu. Rev. Plant Biol..

[17] Slotkin R.K., Freeling M., Lisch D. (2005). Heritable transposon silencing initiated by a naturally occurring transposon inverted duplication. Nat. Genet..

[18] Burgess D., Li H., Zhao M., Kim S.Y., Lisch D. (2020). Silencing of mutator elements in maize involves distinct populations of small RNAs and distinct patterns of DNA methylation. Genetics.

[19] Wang D., Zhang J., Zuo T., Zhao M., Lisch D., Peterson T. (2020). Small RNA-mediated de novo silencing of Ac/ds transposons is initiated by alternative transposition in maize. Genetics.

[20] Zhai J., Bischof S., Wang H., Feng S., Lee T.F., Teng C., Chen X., Park S.Y., Liu L., Gallego-Bartolome J. (2015). A one precursor one siRNA model for Pol IV-dependent siRNA biogenesis. Cell.

[21] Erdmann R.M., Picard C.L. (2020). RNA-Directed DNA methylation. PLoS Genet..

[22] Li Q., Gent J.I., Zynda G., Song J., Makarevitch I., Hirsch C.D., Hirsch C.N., Dawe R.K., Madzima T.F., McGinnis K.M. (2015). RNA-directed DNA methylation enforces boundaries between heterochromatin and euchromatin in the maize genome. Proc. Natl. Acad. Sci. USA.

[23] Kim E.Y., Wang L., Lei Z., Li H., Fan W., Cho J. (2021). Ribosome stalling and SGS3 phase separation prime the epigenetic silencing of transposons. Nativ. Plants.

[24] Cuerda-Gil D., Slotkin R.K. (2016). Non-canonical RNA-directed DNA methylation. Nativ. Plants.

[25] Liu B., Zhao M. (2023). How transposable elements are recognized and epigenetically silenced in plants?. Curr. Opin. Plant Biol..

[26] Singh J., Freeling M., Lisch D. (2008). A position effect on the heritability of epigenetic silencing. PLoS Genet..

[27] Roquis D., Robertson M., Yu L., Thieme M., Julkowska M., Bucher E. (2021). Genomic impact of stress-induced transposable element mobility in Arabidopsis. Nucleic Acids Res..

[28] Guo W., Wang D., Lisch D. (2021). RNA-directed DNA methylation prevents rapid and heritable reversal of transposon silencing under heat stress in *Zea mays*. PLoS Genet..

[29] Ito H., Kim J.M., Matsunaga W., Saze H., Matsui A., Endo T.A., Harukawa Y., Takagi H., Yaegashi H., Masuta Y. (2016). A stress-activated transposon in arabidopsis induces transgenerational abscisic acid insensitivity. Sci. Rep..

[30] Klein S.P., Anderson S.N. (2022). The evolution and function of transposons in epigenetic regulation in response to the environment. Curr. Opin. Plant Biol..

[31] Hirochika H., Sugimoto K., Otsuki Y., Tsugawa H., Kanda M. (1966). Retrotransposons of rice involved in mutations induced by tissue culture. Proc. Natl. Acad. Sci. USA.

[32] Schmitz R.J., Schultz M.D., Lewsey M.G., O’Malley R.C., Urich M.A., Libiger O., Schork N.J., Ecker J.R. (2011). Transgenerational epigenetic instability is a source of novel methylation variants. Science.

[33] Josefsson C., Dilkes B., Comai L. (2006). Parent-dependent loss of gene silencing during interspecies hybridization. Curr. Biol..

[34] Göbel U., Arce A.L., He F., Rico A., Schmitz G., de Meaux J. (2018). Robustness of transposable element regulation but no genomic shock observed in interspecific Arabidopsis hybrids. Genome Biol. Evol..

[35] Yañez-Santos A.M., Paz R.C., Paz-Sepúlveda P.B., Urdampilleta J.D. (2021). Full-length LTR retroelements in Capsicum annuum revealed a few species-specific family bursts with insertional preferences. Chrom. Res..

[36] Zemach A., Kim M.Y., Hsieh P.H., Coleman-Derr D., Eshed-Williams L., Thao K., Harmer S.L., Zilberman D. (2013). The Arabidopsis nucleosome remodeler DDM1 allows DNA methyltransferases to access H1-containing heterochromatin. Cell.

[37] Sigman M.J., Slotkin R.K. (2016). The first rule of plant transposable element silencing: Location, location, location. Plant Cell.

[38] Becker Y. (2000). Evolution of viruses by acquisition of cellular RNA or DNA nucleotide sequences and genes: An introduction. Virus Genes..

[39] Agol V.I., Gmyl A.P. (2010). Viral security proteins: Counteracting host defences. Nat. Rev. Microbiol..

[40] Sasaki T., Kato K., Hosaka A., Fu Y., Toyoda A., Fujiyama A., Tarutani Y., Kakutani T. (2023). Arms race between anti-silencing and RdDM in noncoding regions of transposable elements. EMBO Rep..

[41] Fedoroff N.V. (2013). Molecular genetics and epigenetics of CACTA elements. Methods Mol. Biol..

[42] Duan C.G., Wang X., Xie S., Pan L., Miki D., Tang K., Hsu C.C., Lei M., Zhong Y., Hou Y.J. (2017). A pair of transposon-derived proteins function in a histone acetyltransferase complex for active DNA demethylation. Cell Res..

[43] Vicient C.M., Casacuberta J.M. (2020). Additional ORFs in Plant LTR-Retrotransposons. Front. Plant Sci..

[44] Gómez-Orte E., Vicient C.M., Martínez-Izquierdo J.A. (2013). Grande retrotransposons contain an accessory gene in the unusually long 3’-internal region that encodes a nuclear protein transcribed from its own promoter. Plant Mol. Biol..

[45] Ellstrand N.C. (2014). Is gene flow the most important evolutionary force in plants?. Am. J. Bot..

[46] Mason A.S., Batley J. (2015). Creating new interspecific hybrid and polyploid crops. Trends Biotechnol..

[47] Donoso J.M., Picañol R., Serra O., Howad W., Alegre S., Arús P., Eduardo I. (2016). Exploring almond genetic variability useful for peach improvement: Mapping major genes and QTLs in two interspecific almond × peach populations. Mol. Breed..

[48] Bashir T., Sailer C., Gerber F., Loganathan N., Bhoopalan H., Eichenberger C., Grossniklaus U., Baskar R. (2014). Hybridization alters spontaneous mutation rates in a parent-of-origin-dependent fashion in Arabidopsis. Plant Physiol..

[49] Nieto-Feliner G., Casacuberta J., Wendel J.F. (2020). Genomics of evolutionary novelty in hybrids and polyploids. Front. Genet..

[50] Baack E.J., Whitney K.D., Rieseberg L.H. (2005). Hybridization and genome size evolution: Timing and magnitude of nuclear DNA content increases in Helianthus homoploid hybrid species. New Phytol..

[51] Michalak P. (2009). Epigenetic, transposon and small RNA determinants of hybrid dysfunctions. Heredity.

[52] Comai L., Madlung A., Josefsson C., Tyagi A. (2003). Do the different parental ‘heteromes’ cause genomic shock in newly formed allopolyploids?. Philos. Trans. R. Soc. Lond. B Biol. Sci..

[53] McClintock B. (1984). The significance of responses of the genome to challenge. Science.

[54] Anderson S.N., Stitzer M.C., Zhou P., Ross-Ibarra J., Hirsch C.D., Springer N.M. (2019). Dynamic Patterns of Transcript Abundance of Transposable Element Families in Maize. G3.

[55] Guo B., Chen Y., Zhang G., Xing J., Hu Z., Feng W., Yao Y., Peng H., Du J., Zhang Y. (2013). Comparative proteomic analysis of embryos between a maize hybrid and its parental lines during early stages of seed germination. PLoS ONE.

[56] Barber W.T., Zhang W., Win H., Varala K.K., Dorweiler J.E., Hudson M.E., Moose S.P. (2012). Repeat associated small RNAs vary among parents and following hybridization in maize. Proc. Natl. Acad. Sci. USA.

[57] Liu B., Yang D., Wang D., Liang C., Wang J., Lisch D., Zhao M. (2023). Heritable changes of epialleles in maize can be triggered in the absence of DNA methylation. bioRxiv.

[58] Ungerer M.C., Strakosh S.C., Zhen Y. (2006). Genome expansion in three hybrid sunflower species is associated with retrotransposon proliferation. Curr. Biol..

[59] Ungerer M.C., Strakosh S.C., Stimpson K.M. (2009). Proliferation of Ty3/gypsy-like retrotransposons in hybrid sunflower taxa inferred from phylogenetic data. BMC Biol..

[60] Staton S.E., Ungerer M.C., Moore R.C. (2009). The genomic organization of Ty3/gypsy-like retrotransposons in *Helianthus* (*Asteraceae*) homoploid hybrid species. Am. J. Bot..

[61] Kawakami T., Strakosh S.C., Zhen Y., Ungerer M.C. (2010). Different scales of Ty1/copia-like retrotransposon proliferation in the genomes of three diploid hybrid sunflower species. Heredity.

[62] Kawakami T., Dhakal P., Katterhenry A.N., Heatherington C.A., Ungerer M.C. (2011). Transposable element proliferation and genome expansion are rare in contemporary sunflower hybrid populations despite widespread transcriptional activity of LTR retrotransposons. Genome Biol. Evol..

[63] Ungerer M.C., Kawakami T. (2013). Transcriptional dynamics of LTR retrotransposons in early generation and ancient sunflower hybrids. Genome Biol. Evol..

[64] Renaut S., Rowe H.C., Ungerer M.C., Rieseberg L.H. (2014). Genomics of homoploid hybrid speciation: Diversity and transcriptional activity of long terminal repeat retrotransposons in hybrid sunflowers. Phil. Tran. R. Soc. Lond. Biol. Sci..

[65] Ågren J.A., Huang H.R., Wright S.I. (2016). Transposable element evolution in the allotetraploid *Capsella bursa-pastoris*. Am. J. Bot..

[66] Duan T., Sicard A., Glémin S., Lascoux M. (2023). Expression pattern of resynthesized allotetraploid Capsella is determined by hybridization, not whole-genome duplication. New Phytol..

[67] Senerchia N., Felber F., Parisod C. (2015). Genome reorganization in F1 hybrids uncovers the role of retrotransposons in reproductive isolation. Proc. R. Soc. B. Biol. Sci..

[68] Senerchia N., Felber F., North B., Sarr A., Guadagnuolo R., Parisod C. (2016). Differential introgression and reorganization of retrotransposons in hybrid zones between wild wheats. Mol. Ecol..

[69] Danilova T.V., Akhunova A.R., Akhunov E.D., Friebe B., Gill B.S. (2017). Major structural genomic alterations can be associated with hybrid speciation in *Aegilops markgrafii* (*Triticeae*). Plant J..

[70] Shams I., Raskina O. (2018). Intraspecific and intraorganismal copy number dynamics of retrotransposons and tandem repeat in *Aegilops speltoides Tausch* (*Poaceae*, *Triticeae*). Protoplasma.

[71] Madlung A., Tyagi A.P., Watson B., Jiang H., Kagochi T., Doerge R.W., Martienssen R., Comai L. (2005). Genomic changes in synthetic Arabidopsis polyploids. Plant J..

[72] Madlung A., Henkhaus N., Jurevic L., Kahsai E.A., Bernhard J. (2012). Natural variation and persistent developmental instabilities in geographically diverse accessions of the allopolyploid *Arabidopsis suecica*. Physiol. Plant..

[73] Ha M., Lu J., Tian L., Ramachandran V., Kasschau K.D., Chapman E.J., Carrington J.C., Chen X., Wang X.J., Chen Z.J. (2009). Small RNAs serve as a genetic buffer against genomic shock in Arabidopsis interspecific hybrids and allopolyploids. Proc. Natl. Acad. Sci. USA.

[74] Beaulieu J., Jean M., Belzile F. (2009). The allotetraploid *Arabidopsis thaliana*-*Arabidopsis lyrata* subsp. *petraea* as an alternative model system for the study of polyploidy in plants. Mol. Genet. Genom..

[75] Zhu W., Hu B., Becker C., Doan E.S., Berendzen K.W., Weigel D., Liu C. (2017). Altered chromatin compaction and histone methylation drive non-additive gene expression in an interspecific Arabidopsis hybrid. Genome Biol..

[76] Vaughn M.W., Tanurdzić M., Lippman Z., Jiang H., Carrasquillo R., Rabinowicz P.D., Dedhia N., McCombie W.R., Agier N., Bulski A. (2007). Epigenetic natural variation in *Arabidopsis thaliana*. PLoS Biol..

[77] Li Y., Varala K., Moose S.P., Hudson M.E. (2012). The inheritance pattern of 24 nt siRNA clusters in arabidopsis hybrids is influenced by proximity to transposable elements. PLoS ONE.

[78] Greaves I.K., Groszmann M., Ying H., Taylor J.M., Peacock W.J., Dennis E.S. (2012). Trans chromosomal methylation in Arabidopsis hybrids. Proc. Natl. Acad. Sci. USA.

[79] Groszmann M., Greaves I.K., Albertyn Z.I., Scofield G.N., Peacock W.J., Dennis E.S. (2011). Changes in 24-nt siRNA levels in Arabidopsis hybrids suggest an epigenetic contribution to hybrid vigor. Proc. Natl. Acad. Sci. USA.

[80] Shen H., He H., Li J., Chen W., Wang X., Guo L., Peng Z., He G., Zhong S., Qi Y. (2012). Genome-wide analysis of DNA methylation and gene expression changes in two Arabidopsis ecotypes and their reciprocal hybrids. Plant Cell.

[81] Rigal M., Becker C., Pélissier T., Pogorelcnik R., Devos J., Ikeda Y., Weigel D., Mathieu O. (2016). Epigenome confrontation triggers immediate reprogramming of DNA methylation and transposon silencing in *Arabidopsis thaliana* F1 epihybrids. Proc. Natl. Acad. Sci. USA.

[82] Tang Y., Li X., Hu C., Qiu X., Li J., Li X., Zhu H., Wang J., Sui J., Qiao L. (2022). Identification and characterization of transposable element AhMITE1 in the genomes of cultivated and two wild peanuts. BMC Genom..

[83] Alix K., Heslop-Harrison J.S. (2004). The diversity of retroelements in diploid and allotetraploid Brassica species. Plant Mol. Biol..

[84] Alix K., Joets J., Ryder C.D., Moore J., Barker G.C., Bailey J.P., King G.J., Pat Heslop-Harrison J.S. (2008). The CACTA transposon Bot1 played a major role in Brassica genome divergence and gene proliferation. Plant J..

[85] Shen E., Zou J., Hubertus Behrens F., Chen L., Ye C., Dai S., Li R., Ni M., Jiang X., Qiu J. (2015). Identification, evolution, and expression partitioning of miRNAs in allopolyploid *Brassica napus*. J. Exp. Bot..

[86] Lukens L.N., Pires J.C., Leon E., Vogelzang R., Oslach L., Osborn T. (2006). Patterns of sequence loss and cytosine methylation within a population of newly resynthesized *Brassica napus* allopolyploids. Plant Physiol..

[87] Zou J., Fu D., Gong H., Qian W., Xia W., Pires J.C., Li R., Long Y., Mason A.S., Yang T.J. (2011). De novo genetic variation associated with retrotransposon activation, genomic rearrangements and trait variation in a recombinant inbred line population of Brassica napus derived from interspecific hybridization with *Brassica rapa*. Plant J..

[88] Sarilar V., Palacios P.M., Rousselet A., Ridel C., Falque M., Eber F., Chèvre A.M., Joets J., Brabant P., Alix K. (2013). Allopolyploidy has a moderate impact on restructuring at three contrasting transposable element insertion sites in resynthesized *Brassica napus* allotetraploids. New Phytol..

[89] An Z., Tang Z., Ma B., Mason A.S., Guo Y., Yin J., Gao C., Wei L., Li J., Fu D. (2014). Transposon variation by order during allopolyploidisation between *Brassica oleracea* and *Brassica rapa*. Plant Biol..

[90] Zhang J., Li G., Li H., Pu X., Jiang J., Chai L., Zheng B., Cui C., Yang Z., Zhu Y. (2015). Transcriptome Analysis of Interspecific Hybrid between *Brassica napus* and *B. rapa* Reveals Heterosis for Oil Rape Improvement. Int. J. Genom..

[91] Xu Y., Zhong L., Wu X., Fang X., Wang J. (2009). Rapid alterations of gene expression and cytosine methylation in newly synthesized *Brassica napus* allopolyploids. Planta.

[92] Zhang X., Ge X., Shao Y., Sun G., Li Z. (2013). Genomic change, retrotransposon mobilization and extensive cytosine methylation alteration in *Brassica napus* introgressions from two intertribal hybridizations. PLoS ONE.

[93] Shen Y., Sun S., Hua S., Shen E., Ye C.Y., Cai D., Timko M.P., Zhu Q.H., Fan L. (2017). Analysis of transcriptional and epigenetic changes in hybrid vigor of allopolyploid *Brassica napus* uncovers key roles for small RNAs. Plant J..

[94] Zhang K., Zhang L., Cui Y., Yang Y., Wu J., Liang J., Li X., Zhang X., Zhang Y., Guo Z. (2023). The lack of negative association between TE load and subgenome dominance in synthesized Brassica allotetraploids. Proc. Natl. Acad. Sci. USA.

[95] Zhang M., Liu X.K., Fan W., Yan D.F., Zhong N.S., Gao J.Y., Zhang W.J. (2018). Transcriptome analysis reveals hybridization-induced genome shock in an interspecific F1 hybrid from Camellia. Genome.

[96] Eriksson M.C., Mandáková T., McCann J., Temsch E.M., Chase M.W., Hedrén M., Weiss-Schneeweiss H., Paun O. (2022). Repeat dynamics across timescales: A perspective from sibling allotetraploid marsh orchids (*Dactylorhiza majalis* s.l.). Mol. Biol. Evol..

[97] Zhao T., Tao X., Feng S., Wang L., Hong H., Ma W., Shang G., Guo S., He Y., Zhou B. (2018). LncRNAs in polyploid cotton interspecific hybrids are derived from transposon neofunctionalization. Genome Biol..

[98] Fukai E., Yoshikawa M., Shah N., Sandal N., Miyao A., Ono S., Hirakawa H., Akyol T.Y., Umehara Y., Nonomura K.I. (2022). Widespread and transgenerational retrotransposon activation in inter- and intra-species recombinant inbred populations of *Lotus japonicus*. Plant J..

[99] Petit M., Guidat C., Daniel J., Denis E., Montoriol E., Bui Q.T., Lim K.Y., Kovarik A., Leitch A.R., Grandbastien M.A. (2010). Mobilization of retrotransposons in synthetic allotetraploid tobacco. New Phytol..

[100] Mhiri C., Parisod C., Daniel J., Petit M., Lim K.Y., de Borne F.D., Kovarik A., Leitch A.R., Grandbastien M.A. (2019). Parental transposable element loads influence their dynamics in young *Nicotiana* hybrids and allotetraploids. New Phytol..

[101] Liu B., Wendel J.F. (2000). Retrotransposon activation followed by rapid repression in introgressed rice plants. Genome.

[102] Shan X., Liu Z., Dong Z., Wang Y., Chen Y., Lin X., Long L., Han F., Dong Y., Liu B. (2005). Mobilization of the active MITE transposons mPing and Pong in rice by introgression from wild rice (*Zizania latifolia* Griseb.). Mol. Biol. Evol..

[103] Dong Z.Y., Wang Y.M., Zhang Z.J., Shen Y., Lin X.Y., Ou X.F., Han F.P., Liu B. (2006). Extent and pattern of DNA methylation alteration in rice lines derived from introgressive hybridization of rice and *Zizania latifolia* Griseb. Theor. Appl. Genet..

[104] Wang N., Wang H., Wang H., Zhang D., Wu Y., Ou X., Liu S., Dong Z., Liu B. (2010). Transpositional reactivation of the Dart transposon family in rice lines derived from introgressive hybridization with *Zizania latifolia*. BMC Plant Biol..

[105] Wang H., Chai Y., Chu X., Zhao Y., Wu Y., Zhao J., Ngezahayo F., Xu C., Liu B. (2009). Molecular characterization of a rice mutator-phenotype derived from an incompatible cross-pollination reveals transgenerational mobilization of multiple transposable elements and extensive epigenetic instability. BMC Plant Biol..

[106] Benson C.W., Sheltra M.R., Maughan P.J., Jellen E.N., Robbins M.D., Bushman B.S., Patterson E.L., Hall N.D., Huff D.R. (2023). Homoeologous evolution of the allotetraploid genome of *Poa annua* L. BMC Genom..

[107] Usai G., Mascagni F., Vangelisti A., Giordani T., Ceccarelli M., Cavallini A., Natali L. (2020). Interspecific hybridisation and LTR retrotransposon mobilisation-related structural variation in plants: A case study. Genomics.

[108] Mascagni F., Usai G., Natali L., Cavallini A., Giordani T. (2018). A comparison of methods for LTR-retrotransposon insertion time profiling in the *Populus trichocarpa* genome. Caryologia.

[109] Raza M.A., Yu N., Wang D., Cao L., Gan S., Chen L. (2017). Differential DNA methylation and gene expression in reciprocal hybrids between *Solanum lycopersicum* and *S. pimpinellifolium*. DNA Res..

[110] Gantuz M., Morales A., Bertoldi M.V., Ibañez V.N., Duarte P.F., Marfil C.F., Masuelli R.W. (2021). Hybridization and polyploidization effects on LTR-retrotransposon activation in potato genome. J. Plant Res..

[111] Marfil C.F., Masuelli R.W., Davison J., Comai L. (2006). Genomic instability in *Solanum tuberosum × Solanum kurtzianum* interspecific hybrids. Genome.

[112] Paz R.C., Rendina González A.P., Ferrer M.S., Masuelli R.W. (2015). Short-term hybridisation activates Tnt1 and Tto1 Copia retrotransposons in wild tuber-bearing Solanum species. Plant Biol..

[113] Shaked H., Kashkush K., Ozkan H., Feldman M., Levy A.A. (2001). Sequence elimination and cytosine methylation are rapid and reproducible responses of the genome to wide hybridization and allopolyploidy in wheat. Plant Cell.

[114] Kashkush K., Feldman M., Levy A.A. (2002). Gene loss, silencing and activation in a newly synthesized wheat allotetraploid. Genetics.

[115] Kashkush K., Feldman M., Levy A.A. (2003). Transcriptional activation of retrotransposons alters the expression of adjacent genes in wheat. Nat. Genet..

[116] Banouh M., Armisen D., Bouguennec A., Huneau C., Sow M.D., Pont C., Salse J., Civáň P. (2023). Low impact of polyploidization on the transcriptome of synthetic allohexaploid wheat. BMC Genom..

[117] Kraitshtein Z., Yaakov B., Khasdan V., Kashkush K. (2010). Genetic and epigenetic dynamics of a retrotransposon after allopolyploidization of wheat. Genetics.

[118] Yaakov B., Kashkush K. (2011). Massive alterations of the methylation patterns around DNA transposons in the first four generations of a newly formed wheat allohexaploid. Genome.

[119] Kenan-Eichler M., Leshkowitz D., Tal L., Noor E., Melamed-Bessudo C., Feldman M., Levy A.A. (2011). Wheat hybridization and polyploidization results in deregulation of small RNAs. Genetics.

[120] Kirov I., Dudnikov M., Merkulov P., Shingaliev A., Omarov M., Kolganova E., Sigaeva A., Karlov G., Soloviev A. (2020). Nanopore RNA Sequencing Revealed Long Non-Coding and LTR Retrotransposon-Related RNAs Expressed at Early Stages of Triticale SEED Development. Plants.

[121] Li A., Liu D., Wu J., Zhao X., Hao M., Geng S., Yan J., Jiang X., Zhang L., Wu J. (2014). mRNA and Small RNA Transcriptomes Reveal Insights into Dynamic Homoeolog Regulation of Allopolyploid Heterosis in Nascent Hexaploid Wheat. Plant Cell.

[122] Bento M., Pereira H.S., Rocheta M., Gustafson P., Viegas W., Silva M. (2008). Polyploidization as a retraction force in plant genome evolution: Sequence rearrangements in triticale. PLoS ONE.

[123] Cadle-Davidson M.M., Owens C.L. (2008). Genomic amplification of the Gret1 retroelement in white-fruited accessions of wild vitis and interspecific hybrids. Theor. Appl. Genet..

[124] Heyduk K., McAssey E.V., Grimwood J., Shu S., Schmutz J., McKain M.R., Leebens-Mack J. (2021). Hybridization History and Repetitive Element Content in the Genome of a Homoploid Hybrid, *Yucca gloriosa* (*Asparagaceae*). Front. Plant Sci..

[125] Junaid A., Kumar H., Rao A.R., Patil A.N., Singh N.K., Gaikwad K. (2018). Unravelling the epigenomic interactions between parental inbreds resulting in an altered hybrid methylome in pigeonpea. DNA Res..

[126] Zagorski D., Hartmann M., Bertrand Y.J.K., Paštová L., Slavíková R., Josefiová J., Fehrer J. (2020). Characterization and Dynamics of Repeatomes in Closely Related Species of *Hieracium* (*Asteraceae*) and Their Synthetic and Apomictic Hybrids. Front. Plant Sci..

[127] Edger P.P., Smith R., McKain M.R., Cooley A.M., Vallejo-Marin M., Yuan Y., Bewick A.J., Ji L., Platts A.E., Bowman M.J. (2017). Subgenome dominance in an interspecific hybrid, synthetic allopolyploid, and a 140-year-old naturally established neo-allopolyploid Monkeyflower. Plant Cell.

[128] De Tomás C., Bardil A., Castanera R., Casacuberta J.M., Vicient C.M. (2022). Absence of major epigenetic and transcriptomic changes accompanying an interspecific cross between peach and almond. Hortic. Res..

[129] D’Amico-Willman K.M., Sideli G.M., Allen B.J., Anderson E.S., Gradziel T.M., Fresnedo-Ramírez J. (2022). Identification of Putative Markers of Non-infectious Bud Failure in Almond (*Prunus dulcis* (Mill.) D.A. Webb) through Genome Wide DNA Methylation Profiling and Gene Expression Analysis in an Almond × Peach Hybrid Population. Front. Plant Sci..

[130] Parisod C., Salmon A., Zerjal T., Tenaillon M., Grandbastien M.A., Ainouche M. (2009). Rapid structural and epigenetic reorganization near transposable elements in hybrid and allopolyploid genomes in Spartina. New Phytol..

[131] Giraud D., Lima O., Rousseau-Gueutin M., Salmon A., Aïnouche M. (2021). Gene and Transposable Element Expression Evolution Following Recent and Past Polyploidy Events in Spartina (*Poaceae*). Front. Genet..

[132] Baumel A., Ainouche M., Kalendar R., Schulman A.H. (2002). Retrotransposons and genomic stability in populations of the young allopolyploid species Spartina anglica C.E. Hubbard (*Poaceae*). Mol. Biol. Evol..

[133] Cavé-Radet A., Giraud D., Lima O., El Amrani A., Aïnouche M., Salmon A. (2019). Evolution of small RNA expression following hybridization and allopolyploidization: Insights from Spartina species (*Poaceae*, *Chloridoideae*). Plant Mol. Biol..

[134] Kuo Y.T., Ishii T., Fuchs J., Hsieh W.H., Houben A., Lin Y.R. (2021). The Evolutionary Dynamics of Repetitive DNA and Its Impact on the Genome Diversification in the Genus *Sorghum*. Front. Plant Sci..

[135] De Tomás C. (2023). Transposons, a rich source of genetic variability in crops. Ph.D. Thesis.

[136] Drouin M., Hénault M., Hallin J., Landry C.R. (2021). Testing the Genomic Shock Hypothesis Using Transposable Element Expression in Yeast Hybrids. Front. Fungal Biol..

[137] Kumar S. (2018). Epigenomics of Plant Responses to Environmental Stress. Epigenomes.

[138] Qiu Y., O’Connor C.H., Della Coletta R., Renk J.S., Monnahan P.J., Noshay J.M., Liang Z., Gilbert A., Anderson S.N., McGaugh S.E. (2021). Whole-genome variation of transposable element insertions in a maize diversity panel. G3.

[139] Lee Q.H., Wright S., Bureau T. (2000). Transposon diversity in *Arabidopsis thaliana*. Proc. Natl. Acad. Sci. USA.

[140] Deniz Ö., Frost J.M., Branco M.R. (2019). Regulation of transposable elements by DNA modifications. Nat. Rev. Genet..

